# The Free-and-Easy Style

**Published:** 1843-10

**Authors:** 


*The Free-and-Easy Style.*—The greatest attainments in medical science will not of themselves compensate for the due reserve and decorum which characterise the gentleman in any walk of life, but which is supereminently demanded from members of such a profession as that of medicine. A case in point is afforded by the following anecdote, which, if not true, at least deserves to be so:—A certain physician in Paris, who also filled a professor's chair, having found practice coming to him but slowly, and remarking at the same time that other physicians who were in good practice, were on familiar terms with some of their female patients, suddenly conceived that he had discovered in the latter circumstance the secret of his rivals' success, and resolved to adopt the marks of intimacy on the first fitting occasion. An occasion arrived. He was sent for by a lady of respectability, whom we shall call *Madame Quelq'une*, of the *Rue Joubert*, No. 1000. Having been announced, he walked boldly into the room, and taking his patient by the chin, exclaimed, "Well my dear! and how are you to-day?" The astonished lady promptly replied, "I am very well, Sir, as I will prove to you;" and thereupon rising and ringing the bell, she said to the servant who appeared, "Pay this physician his fees for two visits, but recollect that when he calls again, I am not at home!" The chop-fallen physician left the *Rue Joubert* with a rueful countenance. But the worst was behind. The lady sent for another physician, who was both a gentleman and a witty man, and who, oddly enough, had been a pupil of the professor. After various inquiries, the new medical attendant found it necessary to percuss the chest, an operation which the lady readily permitted, warning her medical adviser at the outset, however, that there was one region, the exploration of which she could by no means permit. Day after day the physician continued his visits, each day he percussed, and he was as constantly reminded of the prohibition. At length his curiosity having been raised quite on tip-toe, he ventured respectfully to inquire which *was* the interdicted region, and to his great surprise learned that it was *the chin.* The patient, who had long been bursting with the secret, now recounted to her Æsculapius the adventure and mishap of his predecessor, and the pupil resolved at some time to read a lesson to his *ci-devant* master- The opportunity very speedily arrived. As he was returning homeward—on foot—he met, surrounded by a posse of pupils, his old preceptor, who, having just left his professorial chair, was about to enter his cabriolet. The professor, thinking this a capital opportunity to inculcate an useful principle into those around him (and perhaps also to show the authority wielded by a physician who kept his cabriolet over one who was obliged to walk), thus addressed his former disciple:—"My dear ——, you seem to have quite forgotten me, and what you owe to my instructions; you never call me in to a consultation!" "Well," said the other, hesitating for an instant, "I recollect. To-morrow, at 2 o'clock, there is to be a consultation, at which I shall be happy if you will assist." "Come, that's hearty!" said the professor, shaking him by the hand, "I begin, after all, to think that you have not quite forgotten your duty. But I had forgotten

(he added, as just about to step into his vehicle) to ask where the consultation is to be held." "At *Madame Quelq'une, Rue Joubert*, No. 1000." The professor vaulted into his cab as if impelled by a rocket, cloaked himself up in a moment, put the horse at the top of his speed, and was off like a shot, next day quite *forgetting* the consultation,—so says the *Gazette des Hôpitaux.—London Lancet*, July 8, 1843.

